# mRNAsi Index: Machine Learning in Mining Lung Adenocarcinoma Stem Cell Biomarkers

**DOI:** 10.3390/genes11030257

**Published:** 2020-02-27

**Authors:** Yitong Zhang, Joseph Ta-Chien Tseng, I-Chia Lien, Fenglan Li, Wei Wu, Hui Li

**Affiliations:** 1 Department of Biochemistry and Molecular Biology, Harbin Medical University, Harbin 150081, China; zhangyitong@hrbmu.edu.cn (Y.Z.); lifl@ems.hrbmu.edu.cn (F.L.); 2 Department of Biochemistry and Molecular Biology, School of Basic Medical Sciences, Beijing Key Laboratory of Tumor Invasion and Metastasis Research, Institute of Cancer Research, Capital Medical University, Beijing 100069, China; 3 Institute of Bioinformatics and Biosignal Transduction, College of Bioscience and Biotechnology, National Cheng Kung University, Tainan 701, Taiwan; tctseng@mail.ncku.edu.tw (J.T.-C.T.); lienichia@i-genomics.com.tw (I.-C.L.); 4 Insight Genomics Inc., National Cheng Kung University, Tainan 701, Taiwan

**Keywords:** lung adenocarcinoma, cancer cell stemness, WGCNA, mRNAsi, machine learning, TCGA

## Abstract

Cancer stem cells (CSCs), characterized by self-renewal and unlimited proliferation, lead to therapeutic resistance in lung cancer. In this study, we aimed to investigate the expressions of stem cell-related genes in lung adenocarcinoma (LUAD). The stemness index based on mRNA expression (mRNAsi) was utilized to analyze LUAD cases in the Cancer Genome Atlas (TCGA). First, mRNAsi was analyzed with differential expressions, survival analysis, clinical stages, and gender in LUADs. Then, the weighted gene co-expression network analysis was performed to discover modules of stemness and key genes. The interplay among the key genes was explored at the transcription and protein levels. The enrichment analysis was performed to annotate the function and pathways of the key genes. The expression levels of key genes were validated in a pan-cancer scale. The pathological stage associated gene expression level and survival probability were also validated. The Gene Expression Omnibus (GEO) database was additionally used for validation. The mRNAsi was significantly upregulated in cancer cases. In general, the mRNAsi score increases according to clinical stages and differs in gender significantly. Lower mRNAsi groups had a better overall survival in major LUADs, within five years. The distinguished modules and key genes were selected according to the correlations to the mRNAsi. Thirteen key genes (CCNB1, BUB1, BUB1B, CDC20, PLK1, TTK, CDC45, ESPL1, CCNA2, MCM6, ORC1, MCM2, and CHEK1) were enriched from the cell cycle Kyoto Encyclopedia of Genes and Genomes (KEGG) pathway, relating to cell proliferation Gene Ontology (GO) terms, as well. Eight of the thirteen genes have been reported to be associated with the CSC characteristics. However, all of them have been previously ignored in LUADs. Their expression increased according to the pathological stages of LUAD, and these genes were clearly upregulated in pan-cancers. In the GEO database, only the tumor necrosis factor receptor associated factor-interacting protein (TRAIP) from the blue module was matched with the stemness microarray data. These key genes were found to have strong correlations as a whole, and could be used as therapeutic targets in the treatment of LUAD, by inhibiting the stemness features.

## 1. Introduction

Lung cancer is the leading fatal malignancy worldwide, causing over 1.5 million cancer-related deaths annually [1,2]; and it is classified into two main subtypes: non-small cell lung cancer (NSCLC, making up 80–85% of all lung cancers) and small-cell lung cancer (SCLC, making up 15–20% of cases) [3,4]. The NSCLCs are histologically divided into three forms: adenocarcinoma (ADC), squamous cell carcinoma (SCC), and large cell carcinoma (LCC) [5,6]. Despite the approximately 20% operable cases of NSCLS and the improvements in chemoradiotherapy, targeted therapy, and immunotherapy, and advances for inoperable cases with the intent to cure, the 5-year survival rates remain low (7–20%), and the recurrence rates remain high at 30–50% [7]. Unlike lung SCC, lung ADC is commonly seen in non-smokers, which inspired us to investigate the uncovered risk factors resulting in lung ADC diagnosis [8].

The rising theory of the cancer stem cell [9,10,11], with features including self-renewal and unlimited proliferation, brings disruption to the diagnosis and treatment of cancer [10]. The widely accepted hypothesis of CSCs, in the field of oncology, suggests that the heterogeneity within a solid tumor follows a hierarchical cell dynamic and the emergence of a small subpopulation of normal somatic stem cells [11]. In this way, the CSC subpopulation is consistently maintained, preserving its characteristics of self-perpetuation and generation of differentiated progeny through asymmetrical division, which gives rise to heterogenic tumors. However, the CSC population has been shown to expand during periods of stress, including chemotherapy treatments, such as cisplatin and 5’fluorouracil, through a symmetrically dividing way. This process makes therapies ineffective for certain kinds of cancers [12]. As CSCs have been found and separated in several carcinomas, including lung cancer in vivo and in vitro [13], CSC research can provide ideas for solving the drug-resistant dilemma of lung cancer.

In the emerging era of immunotherapy, the concept of the tumor microenvironment (TME) [14,15] and immune checkpoint (IC) [16] is vital in developing oncology research. Since the rollout of the first United States (US) Food and Drug Administration (FDA)-approved immune checkpoint inhibitors (ICIs) in 2010, existing ICIs include the following types: (1) anti–cytotoxic T-lymphocyte antigen 4 (CTLA-4) antibodies, including ipilimumab; (2) anti-programmed cell death-1 (anti-PD-1)/programmed cell death ligand-1 (PD-L1) antibodies, including pembrolizumab and nivolumab. Nivolumab, which is approved by the FDA for the treatment of metastatic NSCLC, has, however, been set as a second-line treatment [17]. In addition, the efficacy and application of ICIs for varies subtypes of lung cancer, for example, the brain metastases (BM), is relatively unknown, calling for further study and validation [18].

Machine learning (ML) has been successfully applied in many fields, such as wireless communication, search engines, and speech recognition [19]. For many researchers with a background in medicine and biology, ML can be mysterious, as it often appears together with artificial intelligence (AI), big data, cloud computing, blockchain technology, etc. However, it is a universal concept and method that should be applied in all fields, and especially in medicine and biology. Generally, ML techniques include supervised learning, unsupervised learning, reinforcement learning, (deep) neural networks, and transfer learning. Unsupervised learning, as a bioinformatic method, is most widely used in the field of medical data mining, such as for weighted gene co-expression network analysis (WGCNA) and principal component analysis (PCA) [20].

The basic idea of unsupervised machine learning can be summarized as: To learn a function to describe a hidden structure from unlabeled data. That is to say, input unprocessed raw biological data, such as reads from next-generation sequencing (NGS), data from protein mass spectrometry, and clinical records in epidemiological studies, and output algorithms or modules. In particular, high-throughput technology is expected to achieve a considerable increase in processing biological data and in information rates. Other than the storage and management in untapped databases, high quality, credible and more clinically relevant data mining has been advocated. However, many valuable modules and much information are wasted, as the existing algorithms are powerless in processing and/or utilizing the underlying data [19]. By enhancing the bioinformatics technology from different aspects, these goals can be potentially met.

What is the stemness index based on mRNA expression (mRNAsi) score exactly? Motivated by the rising number of open source databases for medicine in various fields, such as The Cancer Genome Atlas (TCGA), the ML technique is vastly preferable to traditional methods of cancer research. The innovative one-class logistic regression (OCLR) is a kind of unsupervised machine learning algorithm, proposed in 2016, aiming at the advances in cataloging various cell subtypes of tumor biopsies by RNA-sequencing the data, for precision medicine purposes [21]. The OCLR enables the identifications of molecular cell types from a heterogeneous mixture of sub-clones within one-class models, which was proposed to improve on the two earlier established support vector machine (SVM)-based methods.

A promising application for OCLR is to learn the features of subtypes, through deconvolution of high-throughput data, such as RNA-sequencing data, collected from one piece of a tumor biopsy, coexisting with various tumor types. This advantage is useful for the TCGA database, where it is difficult to distinguish the sequencing data of tumor samples coexisting with multiple cell subtypes. Although single-cell sequencing technology is well known for its precise cell subtype division advantage, as the quantity of the original sample is extremely small, more PCR cycles for amplification are required to build the c-DNA library, and the increased cycles will result in accuracy degradation. The OCLR algorithm may be used as a new method for researchers in the field of biocomputing to obtain more indexes, such as “mRNAsi”, standing for precise and rigorous tumor sub-populations at the molecular level.

**Figure 1:**
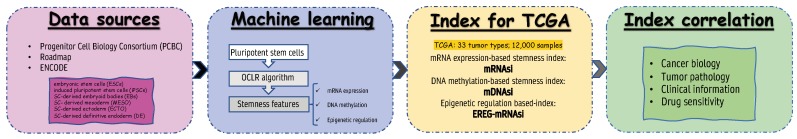
The overall methodology of the Stemness Indexes development [22]. The data sources from the Progenitor Cell Biology Consortium (https://www.synapse.org/pcbc), Roadmap (http://www.roadmapepigenomics.org), and the ENCyclopedia Of DNA Elements (ENCODE) Project (https://www.genome.gov/Funded-Programs-Projects/ENCODE-Project-ENCyclopedia-Of-DNA-Elements) were profiled using the Illumina HumanMethylation 450 (HM450) platform to define stem cell signatures. The pluripotent stem cells include four embryonic stem cells (ESCs), 40 induced pluripotent stem cells (iPSCs), 22 stem cell (SC)-derived embryoid bodies (EBs), 11 SC-derived mesoderm (MESO), 11 SC-derived ectoderm (ECTO), and 11 SC-derived definitive endoderm (DE). The one-class logistic regression (OCLR) algorithm was used to train for stemness features, on stem cell (SC; ESC/iPSC) classes and their differentiated progenitors. As a result, the mRNA expression-based stemness index (mRNAsi), DNA methylation-based stemness index (mDNAsi), and epigenetic regulation based-index (EREG-mRNAsi) were obtained. These stemness indices have also been applied to datasets from TCGA in order to calculate the mRNAsi, EREG-mRNAsi, and mDNAsi scores of the samples. The indexes for each TCGA cases were validated by the correlations with known cancer biology, tumor pathology, clinical information, and drug resistance data.

By applying OCLR to molecular datasets from normal stem cells and their progeny, features of the transcriptome and epigenetics were derived, and indexes representing cancer stemness were produced as a result [22]. To be more specific, the overall methodology for the development of stemness indexes was drawn (Figure 1), including mRNAsi and the epigenetic regulation based-index (EREG-mRNAsi). Moreover, the tumor microenvironment, including immune checkpoint expression and infiltrating immune system cells, was also analyzed for novel cancer stemness. As for the particular tumor’s makeup, the coexistence with either synergistic or antagonistic cells, such as benign cells from the immune system and malignant cells evolved through mutations, reflects a superposition of the contributing cell sub-populations within one tumor biopsy.

As these conditions have been taken into account in the calculation principle of the mRNAsi index, the key genes extracted from our work are associated with the tumor microenvironment. Using the resources of TCGA, researchers have evaluated the stemness of 12,000 cancer samples, covering 33 tumor types. The mRNA expression-based stemness index (mRNAsi index) was considered to represent the mRNA expression-based stemness of the case, and the epigenetic regulation based-index (EREG-mRNAsi) was calculated by the epigenetic regulation features learned by the OCLR algorithm. Higher mRNAsi scores are associated with malignant biological processes in CSCs and more tumor dedifferentiation, according to the histopathological grades. By these stem-cell indexes, we can identify unanticipated biomarkers of lung ADC stem cells, by way of classic and widely known bioinformatic technology.

**Figure 2:**
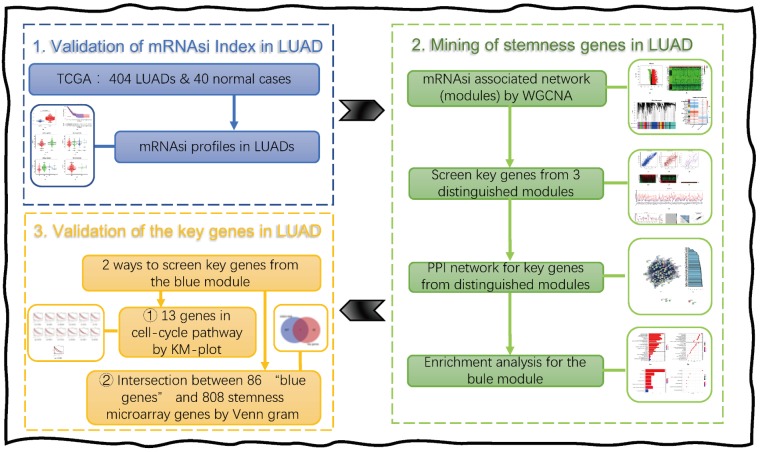
The workflow of this work. Abbreviations are defined as follows, Lung adenocarcinoma (LUAD), the Cancer Genome Atlas (TCGA), Kaplan–Meier-plot (KM-plot), weighted gene co-expression network analysis (WGCNA), and protein–protein interaction (PPI).

In this current work, by taking advantage of both TCGA lung adenocarcinoma (LUAD) cohorts and OCLR algorithm-derived stemness scores, we first extracted a list of stemness associated genes, based on the tumor microenvironment, including the immune checkpoint expression and infiltrating immune system cells and prediction of poor outcomes in LUAD patients. Importantly, we validated these correlations in another LUAD cohort available from TCGA and the Gene Expression Omnibus (GEO) microarray database (Figure 2). In particular, this protocol is universal with all TCGA data sets, suited for cancer stem cell research, including the aspects of the immune checkpoint, and of other cancer types as well.

## 2. Materials and Methods

### 2.1. Software and R Packages

We used the R version 3.6.1 (Action of the Toes) software in this work, on the Windows platform (URL: https://www.r-project.org/). The R packages were all open source and downloaded from the Bioconductor (URL: http://www.bioconductor.org/). Strawberry Perl version-5.14.2.1 (64-bit) was used for data sets merging with a merge script in this work (URL: https://www.perl.org/). All the software was open source and is easily accessed by the URL provided above.

### 2.2. Database and mRNAsi Index

The transcriptome profiling by RNA-sequencing (RNA-seq) of the lung adenocarcinoma set, as well as the information of sex, age, life-status, and stages, were downloaded from the TCGA database (URL: https://tcga-data.nci.nih.gov/tcga/). These data were current as of 5 October 2019. The RNA-seq results of 40 normal samples and 404 tumor samples were combined into a matrix using Perl. Next, the Ensemble database (URL: http://asia.ensembl.org/index.html) was used to convert the Ensemble IDs into official gene names. The microarray (GSE21656) results for validation were downloaded from the Gene Expression Omnibus (GEO) and analyzed online by GEO2R (URL: https://www.ncbi.nlm.nih.gov/geoprofiles/). The mRNAsi index of all of the types of tissue in TCGA was obtained from the attachment for the article by Tathiane M. Malta. We selected the miRNAsi index of lung adenocarcinoma patients and merged this with TCGA data of lung adenocarcinomas, using a Perl merge script, with the unmatched cases deleted. The WilcoxTest was used to investigate the significant difference of mRNAsi between the LUAD subtypes.

### 2.3. Differentially Expressed Genes (DEGs) Analysis

Differentially expressed analysis was performed using the package “limma”, and the Wilcox Test was used in the processing. A Foldchange > 1 and adj.p (false discovery rate, FDR) < 0.05 were considered to be the cut-offs to screen for DEGs between lung adenocarcinoma and normal sets. The heat-map and volcano plot was drawn by R using the package “pheatmap”. The box-plots of the key genes for validation were plotted by R, using the package “ggpubr”. The Multiple Gene Comparison was drawn on GEPIA (http://gepia.cancer-pku.cn/index.html) [23], a web server for cancer and normal gene expression profiling and interactive analyses. We set the log-scale parameter as $\log_{2}\left(TPM+1\right)$ to transform the expression data for plotting. “TCGA tumor + TCGA normal + GTEx normal” was selected for plotting the matched normal data. The Pathological Stage plots for key genes in LUADs were analyzed on GEPIA. We chose $\log_{2}\left(TPM+1\right)$ to transform the expression data, using major stage, for plotting. The method for differential gene expression analysis is one-way ANOVA, using the pathological stage as a variable for calculating differential expression. Pr(>F) < 0.05 was considered statistically significant.

### 2.4. Overall Survival Curve

To determine the prognostic value of mRNAsi scores, we drew Kaplan–Meier plots of the mRNAsi index to explore the difference in overall survival between patients with low and high mRNAsi index. The R packages “survival” and “surviminer” were used for this part, and the relationship was tested by the log-rank. As for the validation of the key genes, Kaplan–Meier survival curves of the key genes were drawn by the online tool Kaplan–Meier plotter (URL: http://www.kmplot.com/analysis/index.php?p=service) [24].

### 2.5. The WGCNA Analysis to Filter Key Genes

The clustering was performed using WGCNA, and the module-trait correlations with mRNAsi and EREG-mRNAsi were plotted by R software. The R packages “matrixStats”, “Hmisc”, “foreach”, “doParallel”, “fastcluster”, “dynamicTreeCut”, “survival”, and “WGCNA [25]” were used in this section. We processed the following protocol to prepare the input data. First, we deleted the normal data set and the cases with missing data (Appendix A). Then, we clustered according to the gene expression level of the samples, reducing the outlier (Appendix A). After the intersection with the mRNAsi data, the prepared input was used in the following analysis.

The power-value was selected to build up a scale-free network, according to the Pearson correlation coefficient among genes, which was determined equal to 4 by calculating the correlation gene between the scale-free $R^{2}$ and gene main connectivity (Appendix A). A GeneTree was constructed according to the power-value, and the dynamic module was identified with a minimum size of 50 genes (Appendix A). Close modules were merged at the Diss Thres of 0.25 (Appendix A). We selected three modules with the highest correlation of mRNAsi, and performed correlation analysis for each merged module, filtering out the key genes correlating with both the module and mRNAsi index.

### 2.6. Gene Co-Expression Analysis

The co-expression relationships between key genes within a module were calculated based on the gene expression levels, to investigate the strength of these relationships at the transcriptional level. The R “corrplot” package was used to compute the Pearson correlations between genes. The correlation between MSRB3 and PRKG1 was profiled on LinkedOmics (http://www.linkedomics.org/login.php) [26]. The LUAD dataset was selected from TCGA for analysis, and the data were analyzed by the Pearson Correlation test. The results with a correlation coefficient > 0.3 and *p*-value < 0.01 were considered statistically significant.

### 2.7. Construction of Protein–Protein Interaction (PPI) Network

The PPI network was retrieved from STRING Version 11.0 (URL: https://string-db.org/) [27], and the bar plot is shown for nodes in the network calculated to have top connectivity. The minimum required interaction score was set at 0.4 of medium confidence, and we disconnected nodes in the network that are hidden. We calculated the number of adjacent nodes of each gene in the PPI network, and sorted the genes according to the number of adjacent nodes by a bar-plot.

### 2.8. Enrichment Analysis of DEGs

Gene Ontology (GO) functional enrichment and Kyoto Encyclopedia of Genes and Genomes (KEGG) pathway enrichment were performed using the R packages “clusterProfiler”, “enrichplot”, and “ggplot2” (*p*-value < 0.05, *q* value < 0.05). The R package “org.Hs.eg.db”, also described as Genome-wide annotation for Humans, was used for mapping the key genes with the Ensemble ID. The bar-plot and the bubble-plot were put out by R, for visualizing the top results.

### 2.9. Venn Diagrams

A Venn diagram was used to calculate the intersections of the list of key genes and differential expression genes from GSE21656, via the online tool Draw Venn Diagram (URL: http://bioinformatics.psb.ugent.be/webtools/Venn/).

## 3. Results

### 3.1. mRNAsi Index Is Significantly Associated with Lung Adenocarcinoma

**Figure 3:**
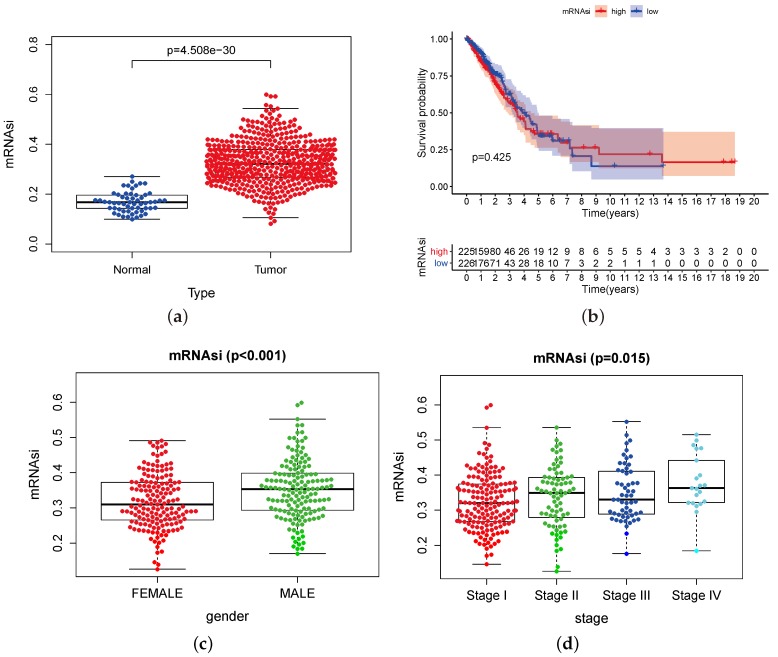

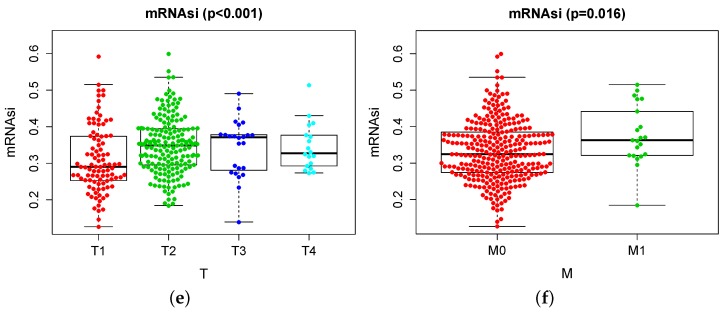
The correlation of global mRNAsi profiles with LUAD subtypes. (**a**) the scatter plot shows that the mRNAsi index expression in 404 tumor cases is higher than that in 40 normal cases (*p*-value < 0.001); (**b**) the LUAD cases were divided into two groups based on their mRNAsi scores: the top half of 225 cases with higher mRNAsi scores and the bottom half of 226 cases with lower mRNAsi scores. The Kaplan–Meier survival curve shows that the median survival of the low score group is longer in the first five years. Few of both groups survived more than five years, but the high group enjoyed a higher survival probability. However, it is not statistically different as a whole, by the Log-rank test *p*-value = 0.425; (**c**) the distribution of mRNAsi scores for genders of LUAD cases. The box-plot shows that there is a significant association between gender and mRNAsi scores; mRNAsi expression is higher in male cases than female (*n* = 404, *p*-value < 0.001); (**d**) the distribution of the mRNAsi index for the clinical stage of LUAD cases. The mRNAsi scores increase in more advanced clinical stages, and extremely so in stageIV (*p*-value = 0.015). (**e**,**f**) The distribution of the mRNAsi index for the TNM degree of LUAD cases; few unknown cases were deleted out. The mRNAsi score increases in T1–T3 degree and declines in T4 (*p*-value < 0.001); group of M1 degree have a higher mRNAsi score than M0 (*p*-value = 0.016).

We downloaded the transcriptome profiling for the gene expression and clinical information of 404 LUAD cases and 40 normal cases from the TCGA database. The information contains the gender, age, life-status, survival time, disease stage, and Tumor-Node-Metastasis (TNM) stage classification, and unknown information was deleted before analysis. The mRNAsi index of each case was downloaded from the appendix of Malta’s work [22] and merged with the TCGA dataset. Based on the OCLR algorithm, the mRNAsi and EREG-mRNAsi scores ranged from 0 to 1, stemless and stemness, respectively. In our study, the mRNAsi is compared in different aspects between the normal and the tumor group, low and high mRNAsi index group, and different subtypes. The mRNAsi score is higher in the tumor group than in the normal group (Figure 3a), which generally indicates the significance of mRNAsi in lung ADC.

We divided the 404 LUAD cases into high and low groups based on their mRNAsi scores and drew the Kaplan–Meier (K–M) survival curves (Figure 3b) to find out the potential correlation of overall survival with high mRNAsi scores and/or low mRNAsi scores. Overall, the K–M survival curves are not statistically significant. Interestingly, however, the high and low curves showed a dramatic intersection right at the point of the fifth year. The 5-year survival rate of lung cancer remains low, thus most cases survive in our study within five years. In the first five years, the survival probability of high mRNAsi index cases is less than the low of the index case, and the following 2 years see an essential flat of the survival probability curves. The cases with high mRNAsi scores survived more than seven years, and, in rare cases, they lived longer, and enjoyed an even higher survival probability.

To reveal the correlation of global mRNAsi profiles with clinical features, we drew scatter plots, as follows. In terms of gender (Figure 3c), in the cases we studied, the mRNAsi index of males was higher than that of females (*p*-value < 0.001). According to the clinical stage of the cases, we found that the early-stage lung cancer (stage I) mRNAsi score was lower than the middle and advanced stage (stage II–IV) LUAD group (Figure 3d), although there was a small decrease in the stage III lung cancer group (*p*-value = 0.015, < 0.05). Grouped by TNM staging, T and M staging were statistically significant. The T stage represents the size of the tumor (Figure 3e). Compared with the T1 group, the mRNAsi scores of the T2 and T3 groups were significantly increased. Although the mRNAsi value of the T4 group decreased, the median number was still higher than that of the T1 group (*p*-value < 0.001). The M stage represents whether the tumor has distant metastasis (Figure 3f). The mRNAsi index of the M1 group is higher than that of the M0 group (*p*-value = 0.016).

### 3.2. The Screening of DEGs and Construction of the WGCNA Co-Expression Network

**Figure 4:**
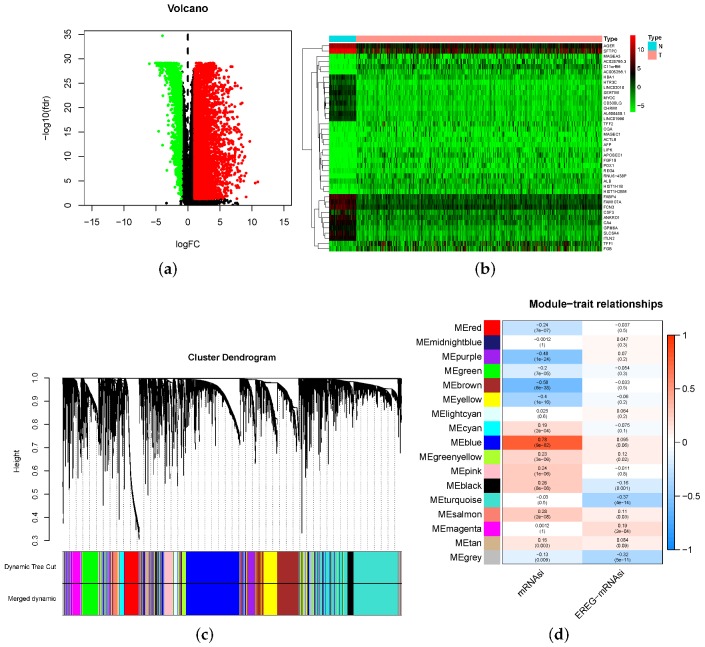
The mRNAsi index associated weighted gene co-expression network analysis (WGCNA) of lung adenocarcinoma. (**a**,**b**) differentially expressed genes; green indicates downregulated genes and red indicates upregulated genes. The Wilcox test shows that there are 500 genes downregulated and 2876 genes upregulated, false discovery rate (FDR) < 0.05 and |logFC| > 1 are considered meaningful. (**c**) The samples with a deflection of gene expression were cut off, and the appropriate power value was determined (=4) as a soft threshold, taking both the scale-free correlation coefficient and mean connectivity into consideration. Then, a co-expression module in LUAD was identified using the power value (Appendix A). Finally, the dendrogram was drawn with branches of the cluster corresponding to the 17 different gene modules, and modules with a standard of the minimum size of 50 genes for module merging were screened out; (**d**) the correlation between the gene modules and clinical traits, including both mRNAsi and EREG-mRNAsi. The correlation coefficient in the heat-map is equal to the correlation between each gene module and the clinical traits, which decreased from red to blue, also annotated with the corresponding *p*-value.

To construct a gene co-expression network of lung ADC in a more targeted manner, we first screened DEGs in data sets of 40 normal cases and 404 lung ADC cases. The sequencing data were filtered, normalized, and differentially analyzed to compare lung ADC and normal cases from TCGA. From this analysis, 3376 DEGs were screened out, of which 500 were upregulated, and 2876 were downregulated (Figure 4a). We also extracted the top 20 DEGs that up- and downregulated and demonstrated this as a heat-map (Figure 4b).

**Figure A1:**
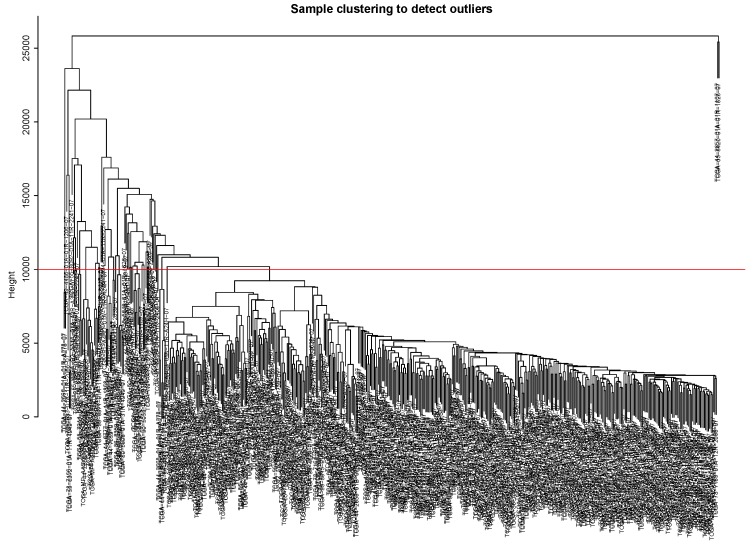
The samples over the red line were considered as the deflection of gene expression, and they were deleted for the outlier elimination.

**Figure A2:**
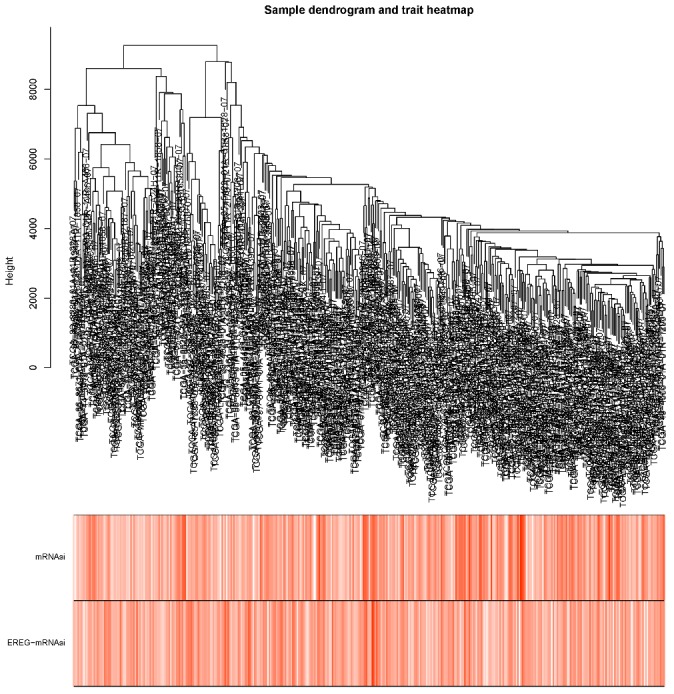
The heat-map of the general data showed an overview of the mRNAsi values and the EREG-mRNAsi values.

We then performed the WGCNA co-expression network analysis of the filtered DEGs. Some of the details are shown in Appendix A to avoid disrupting the flow of the main text. First, the samples with the deflection of gene expression were deleted for the outlier elimination (Figure A1). Next, we made a heat-map of the general data, to show an overview of the mRNAsi values and the EREG-mRNAsi values (Figure A2).

**Figure A3:**
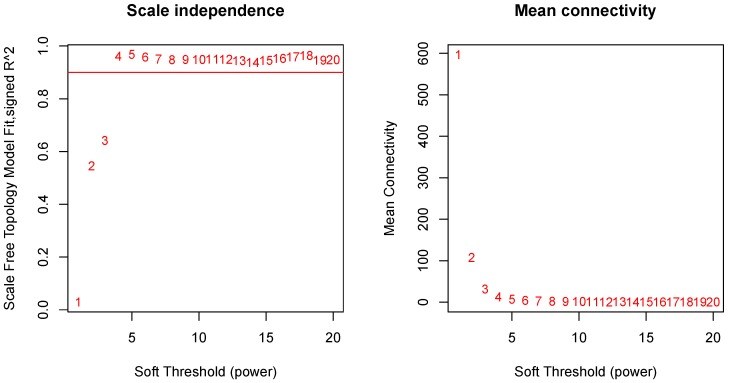
Considering the correlation coefficient $R^{2}$ in the scale-free network fitting process and the mean connectivity in the scale-free network model, we selected 4 as the power value.

**Figure A4:**
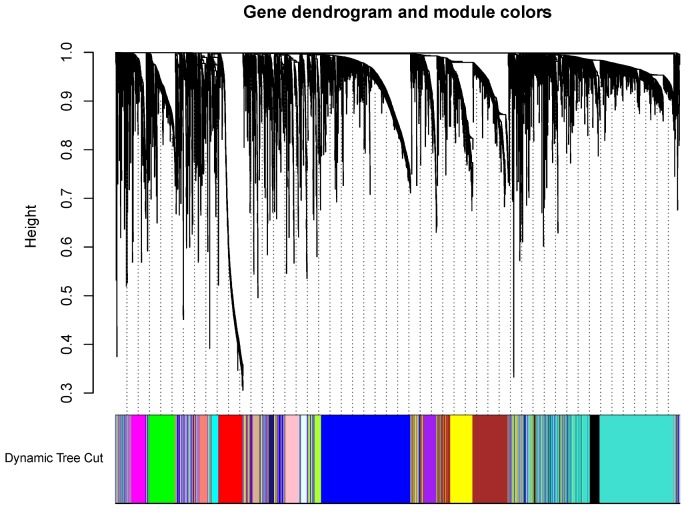
Clustering the DEGs as gene-trees.

**Figure A5:**
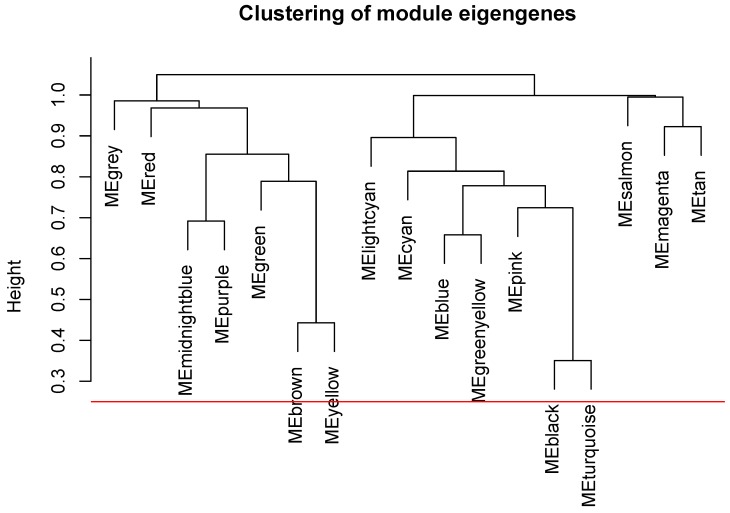
Calculating the module similarity to merge the modules with high similarity.

A scale-free network means that, in a complex system, although most of the nodes have only a few related nodes, a few distinct nodes have connections of up to several million. Any network with this feature can be called a scale-free network. In many systems, there are scale-free structures: for example, the World Wide Web, social networks, and commercial fields, including the field of biology. Since the scale-free network follows the Power-law, we consider the correlation coefficient $R^{2}$ in the scale-free network fitting process and the mean connectivity in the scale-free network model, selecting 4 as the power value (Figure A3). Using the power value, we clustered the DEGs as gene-trees and cut the trees into modules with a minimum size of 50 genes (Figure A4). We calculated the similarity of the modules (Figure A5) and then merged the modules with high similarity (Figure 4c).

For the convenience of the description, we named these modules in color. To show the correlations between each module and the stemness traits, we drew a heat-map as an overview (Figure 4d). According to the mRNAsi correlation coefficient $R^{2}$, we choose blue ($R^{2}$ = 0.78), brown ($R^{2}$ = −0.58), and purple ($R^{2}$ = −0.48) as the target modules, for the possible potential links with stemness features in LUAD.

### 3.3. Extracting and Profile of the Key Genes

**Figure 5:**
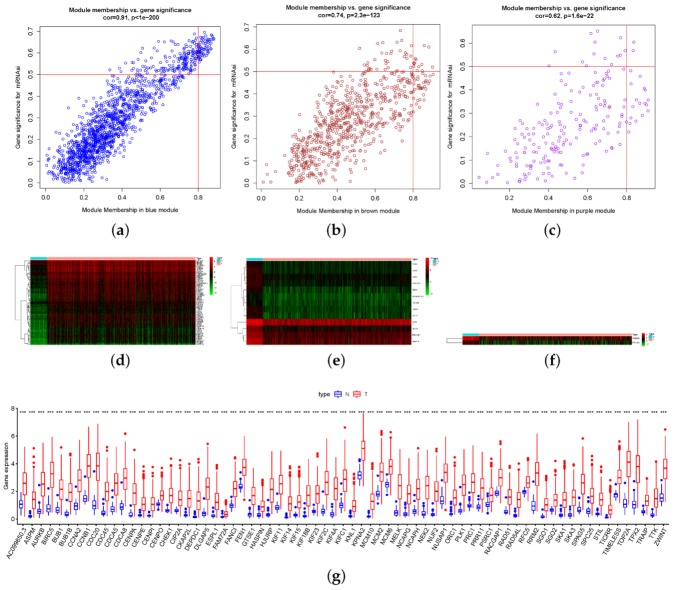

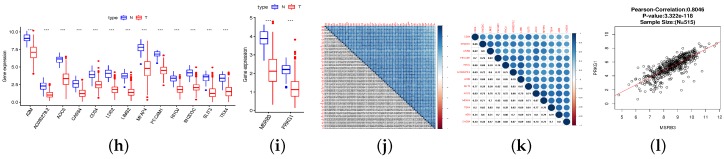
The selection of the key genes from the blue, brown, and purple modules. (**a**–**c**) The dots in the upper right quadrant of the scatter plot represent the key genes we screened. Each scatter stands for a gene, which have two score possibilities: one is its correlation with the module—the module membership (MM) score, the other is its association with the mRNAsi index—the gene significance (GS) score. The standard for screen key genes was set as both MM > 0.8 and GS > 0.5 simultaneously, which represents that this gene has both high stem cell properties and a central position in its module. The profile of key gene expression. (**d**–**f**) The heat-maps show the global differential expression of genes between normal and tumor cases in the LUAD dataset of TCGA in each module; green indicates downregulated genes, and red indicates upregulated genes. (**g**–**i**) The box-plots show that all the key genes have statistically significant differential expression between normal and tumor cases in the LUAD dataset of TCGA, specifically. The correlation among key genes of each module at the transcriptional level. (**j**,**k**) The upper part of the figures show the degree of positive correlation according to the color, and the lower part is the representation of the corresponding correlation value (Pearson correlation coefficient, R > 0.3). (**l**) The correlation plot of the only two genes in the purple module shows a strong correlation (Pearson correlation coefficient, R = 0.8064).

The blue module is positively correlated with the mRNAsi index, which has 1500 genes. The purple and brown modules were negatively correlated with the mRNAsi index. The purple and brown modules have 199 and 706 genes, respectively. The module membership (MM) score stands for the gene’s correlation with its module, and the gene significance (GS) score represents the gene’s correlation with the mRNAsi index. To further narrow down the range of key genes, we screened each gene by two standards: MM > 0.8 and GS > 0.5 (Figure 5a–c). In units of each screened module, we listed heat-maps (Figure 5d–f) and the box-plots (Figure 5g–i) to show the different expressions of the key genes between the normal and lung ADC cases in TCGA. The positively correlated gene in the blue module were upregulated in tumor cases, and the negative brown and purple genes were downregulated in tumor cases, which preliminarily confirmed the stemness of these key genes.

We also performed correlation analyses of the key gene expression in the TCGA lung ADC dataset, which verifies the relevance of the genes within each module. To display the blue and brown modules as a whole, we draw the correlation plot as follows: the upper part of the figure shows the degree of correlation according to the color, and the lower part is the representation of the corresponding correlation value (Figure 5g,h). As there are only two genes in the purple module, we show the results more specifically as follows (Figure 5i). The screened genes in each module are positively correlated.

### 3.4. Protein–Protein Interactions (PPI) among Genes of Each Module

**Figure 6:**
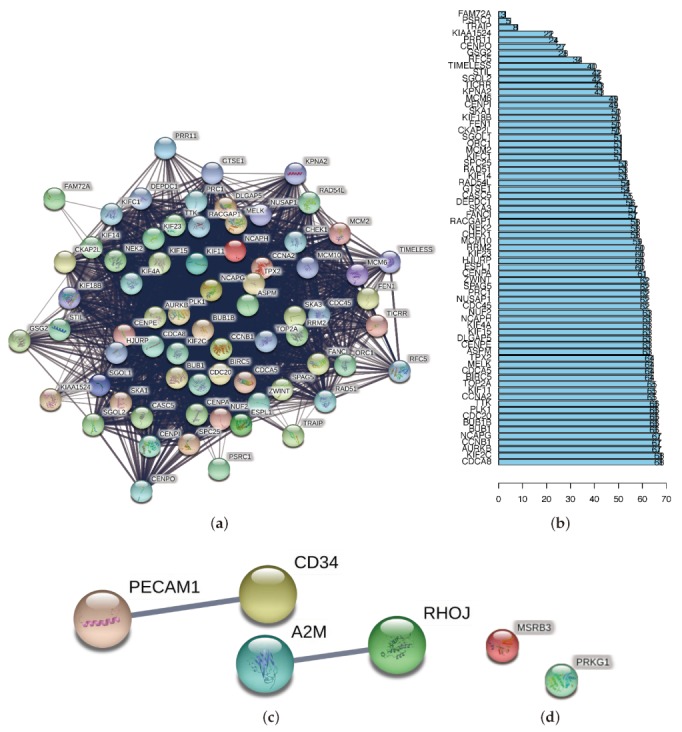
(**a**,**c**,**d**) PPI networks were drawn using the online tool STRING (URL: https://string-db.org/), for the key proteins from the blue, brown, and purple modules. We set the minimum required interaction score as medium confidence (0.4) and hid disconnected nodes in the network. (**b**) As the blue module had the most distinguished scale, we profiled a bar-plot to list its key genes by the counts of connections.

To better explore the interplay among the key genes, we developed protein–protein interaction networks of each module, using the online tool STRING (URL: https://string-db.org/). In the blue module (Figure 6a), 70 nodes and a vast 1879 edges were formed in the network, and the PPI enrichment *p*-value: <1.0 × 10^−16^. Much more than the expected number of edges (118) shows that these genes have more interactions among themselves than what would be expected for a random set of proteins of similar size, drawn from the genome. Such an enrichment indicates that the key genes are at least partially biologically connected as a group. Additionally, remarkable nodes are shown in the bar-plot (Figure 6b), in order to choose the genes that have the most connections with other members of the module. In the brown module (Figure 6c), 12 input nodes formed four nodes and two edges in the network. However, the network does not have significantly more interactions than expected (the expected number of edges = 1, PPI enrichment *p*-value = 0.149). For the purple module (Figure 6d), the only two nodes-MSRB3 and PRKG1 have no direct interaction.

### 3.5. Functional Enrichment Analysis of Key Genes

**Figure 7:**
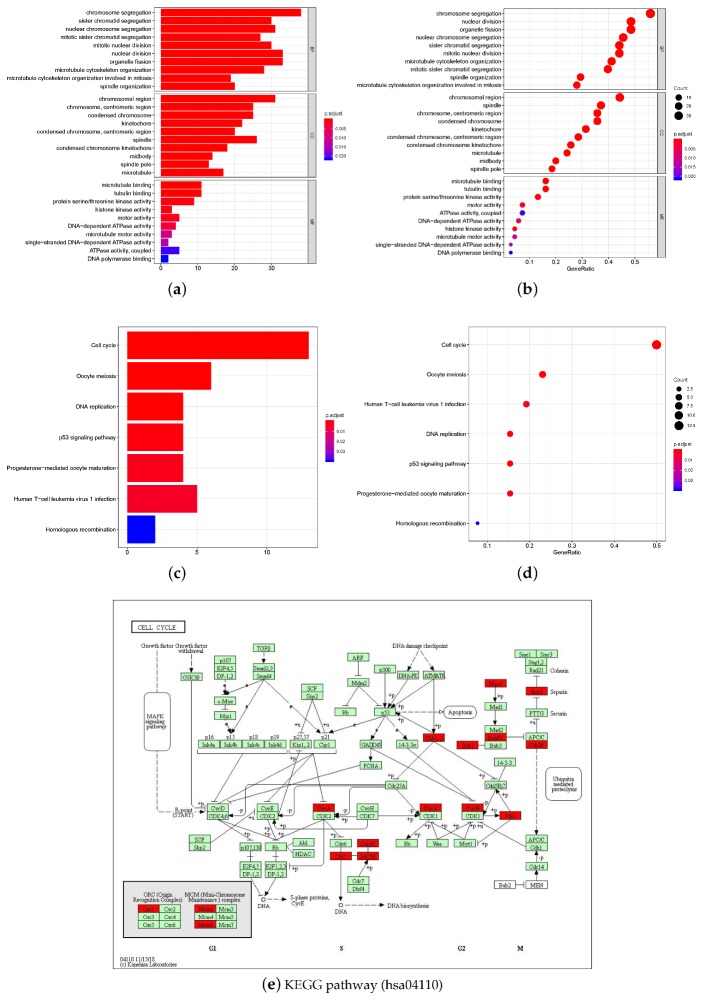
The Gene Ontology (GO) term and Kyoto Encyclopedia of Genes and Genomes (KEGG) pathway analysis for key genes from the blue module. The significant terms and pathways are represented by the negative decadic logarithm of the enrichment adjusted *p*-value (the longer bars indicate more significant clusters). (**a**,**b**) The GO analysis was done in blue modules; bar-plots and bubble-plots are drawn to show the top terms in groups of biological process (BP), cellular component (CC), and molecular function (MF). (**c**,**d**) The top terms of the KEGG pathway analysis for genes in the blue module are shown by bar-plot and bubble-plot also. (**e**) The cell cycle pathway (KEGG: hsa04110) was performed by DAVID (URL: https://david.ncifcrf.gov/tools.jsp), and key genes are marked in red.

To show the functional association of the blue module genes, we used the “clusterProfiler” package for gene enrichment. The GO analyses revealed that the principal biological functions of the blue module were chromosome segregation, chromosomal region, and microtubule-binding, which were mainly associated with the cell cycle and chemo-resistance (Figure 7a,b). We performed KEGG analysis within the blue module, and the most distinguished pathway was also related to the cell cycle (Figure 7c,d). To further illustrate the bio-function of the 13 genes, we showed the KEGG cell cycle pathway (hsa 04110), with the stemness genes marked in red. The cell cycle-related proteins and their positions in the pathway may be related to lung adenocarcinoma stem cell-related mechanisms (Figure 7e).

We found it impressive that the terms on microtubules are also involved in the stemness study. The dynamics of the microtubules are associated with the grading of malignancy and prognosis of cancer tissues [28]. In our previous research on drug resistance of lung adenocarcinoma, we have found that microtubules can be a target for sulforaphane (SFN) to reduce the drug resistance of paclitaxel (PTX), which agrees well with the above analysis [29].

### 3.6. Validation of Key Genes

**Figure 8:**
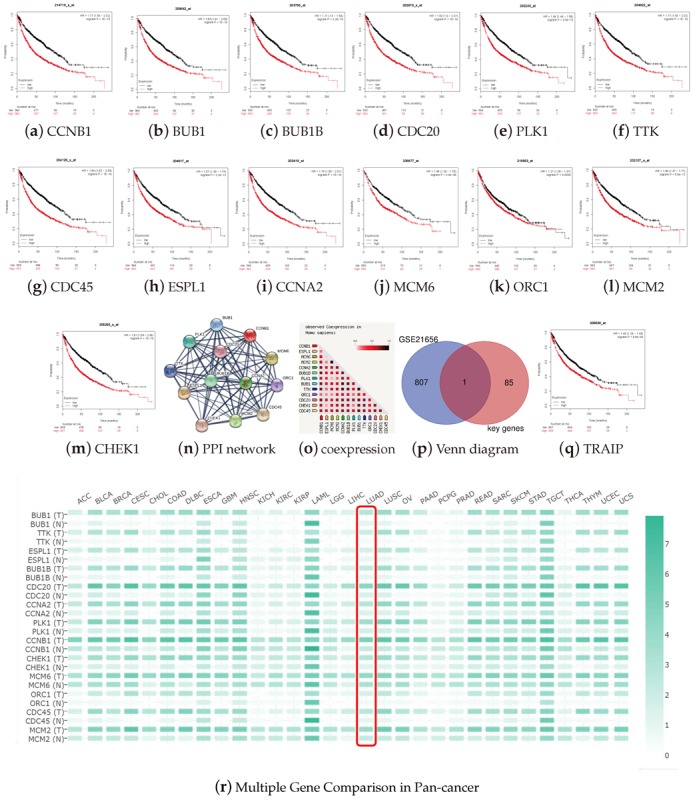
Validation of the key genes. Kaplan–Meier survival plots (K–M plot) were generated using the online tool, Kaplan–Meier plotter. The log-rank test was used, and a *p*-value < 0.001 was considered as a significant difference. (**a**–**m**) There are 13 cell cycle pathway genes enriched from the blue module. K–M plots were drawn to profile a gene’s prognostic value in LUAD, by the comparison of survival probability between the set of high-expression (Red curve) and the low (Black curve). The results show that the 13 key genes are related to poor outcome in LUADs; (**n**) the PPI network of 13 cell cycle genes performed by STRING, enrichment *p*-value < 1.0 × 10^−16^. (**o**) Co-expression predicts a functional association. The co-expression scores are based on RNA expression patterns and protein co-regulation, provided by ProteomeHD. In the triangle-matrices above, the intensity of color indicates the level of confidence that the two proteins are functionally associated, given the overall expression data in the organism. (**p**) A Venn diagram was drawn for mapping stemness microarray GSE21656 and 86 key genes from the blue module. The intersection set within only one gene was tumor necrosis factor receptor associated factor-interacting protein (TRAIP). (**q**) The K–M plot of TRAIP is related to the poor outcome; (**r**) the mRNA expression comparison of key genes in pan-cancer. The expression matrix plots of key genes were analyzed on the web server gene expression profiling and interactive analyses (GEPIA), showing the mRNA expression difference between tumor and normal cases. The density of color in each block represents the median expression value of the target gene, normalized by the maximum median expression value across all blocks. Different genes in the same tumors or normal tissues can be compared within this plot.

To validate the prognostic value of the key genes, we performed the overall survival curves (Figure 8a–m). As the blue module was positively correlated with mRNAsi, blue genes represented the CSC features. Higher gene expression of the 13 cell cycle pathway genes were associated with poorer overall survival in LUADs, *p*-value < 0.01. To verify the relationships at the protein level, we drew the PPI network (Figure 8n) and co-regulation map (Figure 8o) for the human proteome, for a deeper identification of protein functions by ProteomeHD (URL: www.proteomeHD.net) [30].

The PPI cluster with 13 nodes and 78 edges has significantly more interactions than the expected number of nine edges, and the co-regulation results indicate a high level of confidence that the clustered set of 13 proteins are functionally associated as well. To overview the expressions of the key genes in the pan-cancer, we performed the expression matrix of the key genes. By Multiple Gene Comparison between tumor and normal cases using GEPIA, we found that these genes were upregulated not only in LUAD, but also in the pan-cancer scale. (Figure 8r). This implies that the stem cell properties of these key genes may be universal. To further explore the key genes, we also analyzed the expression of key genes with the pathological tumor stage in LUADs using GEPIA. The violin plots showed that the expressions of the key genes were significantly upregulated according to the stage (Appendix B).

We also tried to validate the key genes by microarray. The data of GSE21656 was downloaded from the GEO database, and the online tool GEO2R extracted the DEGs. This microarray was used to profile cisplatin-resistant lung cancer cells (CDDP-R) versus their parental cells, to investigate the CSCs hypothesis of chemo-radiation resistance [31]. A Venn diagram was used to map GSE21656 with 86 key genes screened from the blue module by GS and MM scores. TRAIP (TRAF-interacting protein) was found to be the co-DEG (Figure 8p). The survival analysis of TRAIP reflected its prognostic value in clinical data (Figure 8q).

## 4. Discussion

The morbidity and mortality of lung cancer remains high worldwide. As a major subtype of NSCLC, the risk factor of LUAD is not as clear as lung SCC. Regarding LUAD’s targeted treatment strategies, such as EGFR KRAS, ALK, etc., druggable genetic alterations are crucial for their correlation with clinical and pathological features. These decisions about therapeutic strategies affect the prognosis of lung adenocarcinoma patients [32,33,34]. For example, EGFR and KRAS mutated LUADs have a dramatic prognosis among non-smokers and smokers. Another example is the resistance to EGFR-targeted drugs caused by ALK mutations. In recent years, CSCs have been reported to be closely related to cancer recurrence, metastasis, and chemo-resistance, inducing high mortality [35,36].

The morbidity and mortality of lung cancer remains high worldwide. As a major subtype of NSCLC, the risk factor of LUAD is not as clear as lung SCC. Regarding LUAD’s targeted treatment strategies, such as EGFR KRAS, ALK, etc., druggable genetic alterations are crucial for their correlation with clinical and pathological features. These decisions about therapeutic strategies affect the prognosis of lung adenocarcinoma patients [32,33,34]. For example, EGFR and KRAS mutated LUADs have a dramatic prognosis among non-smokers and smokers. Another example is the resistance to EGFR-targeted drugs caused by ALK mutations. In recent years, CSCs have been reported to be closely related to cancer recurrence, metastasis, and chemo-resistance, inducing high mortality [35,36].

The morbidity and mortality of lung cancer remains high worldwide. As a major subtype of NSCLC, the risk factor of LUAD is not as clear as lung SCC. Regarding LUAD’s targeted treatment strategies, such as EGFR KRAS, ALK, etc., druggable genetic alterations are crucial for their correlation with clinical and pathological features. These decisions about therapeutic strategies affect the prognosis of lung adenocarcinoma patients [32,33,34]. For example, EGFR and KRAS mutated LUADs have a dramatic prognosis among non-smokers and smokers. Another example is the resistance to EGFR-targeted drugs caused by ALK mutations. In recent years, CSCs have been reported to be closely related to cancer recurrence, metastasis, and chemo-resistance, inducing high mortality [35,36].

Research on the therapeutic targeting of LUAD stem cells is very urgent. Therefore, a comprehensive study cohort design, including a comparison of stem cell-related genes across all molecular subtypes, may help a lot in this innovative scientific perspective. In addition, detecting the emergence of these druggable genetic alterations in pan-cancer cases, and whether there are changes in the expression of the same mRNAsi-related genes, is also a question worthy of discussion in future work. The mother set with all the RNA-seq data mixing various mutations modeled in this work can be divided into several child sets by their genetic alterations, i.e., Subset EGFR, Subset KRAS, Subset ALK, etc.

In the current study, we attempted to find key genes related to lung ADC stem cells in TCGA database. The key genes identified in this work make broad sense over the whole case; however, they have relatively loose molecular classification constraints. Therefore, the prognosis value for precision medicine is a large concern. To address this issue, in our next work, we plan to redesign the cohort to explore the key genes within each molecular subtype separately, as the cases therein would embrace more practical constraints. Before our next work, we first need to achieve bounds for the data-mining made within the molecular subsets.

The deprivation of differentiated phenotypes and the acquisition of stemness characteristics are manifestations of cancer progression. In this study, we focused on the key genes correlated to stemness features using WGCNA based on an mRNAsi index. This index was calculated by the OCLR algorithm. The tumor case had a higher mRNAsi score compared with the normal case. The mRNAsi scores increased with the disease stage and the TNM stage of LUAD, although there was a small fall in disease stage III and stage T4. On the whole, the mRNAsi scores of LUAD increased with the stages, and a small drop in individual groups may be related to insufficient sample size. The high mRNAsi group showed a lower survival probability than the low group in the first five years, which was consistent with the poor outcome associated with stemness features. However, after most of the cases died, the rare cases with higher mRNAsi scores survived for more than five years in LUADs.

It is well worth exploring whether the rare ones have uncovered mutations related to cancer stem cells in LUADs. According to the clinical data, we found that male patients had higher mRNAsi scores. Although the difference in sex between mRNAsi needs further verification, determining whether genetic factors or acquired factors caused this is also worth studying, by taking advantage of the short tandem repeat (STR) analysis technique [37].

We constructed co-expression modules through WGCNA and selected three modules (blue, brown, and purple) with the greatest correlations with mRNAsi. Key genes were screened from the blue module based on the GS and MM scores. The expression of key genes from mRNAsi correlated modules were differentially regulated in LUADs accordingly. There were strong co-expression relationships at the transcript level, within each module. There was also a strong PPI network among proteins of the blue module. However, only two pairs from the brown had a PPI relationship, and two purple genes did not have a direct interaction at the protein level. This indicates that these two sets of proteins are either rather small or essentially a random collection.

Fortunately, this does not necessarily mean that it is not a biologically meaningful selection of proteins. It may simply be due to a lack of study of these genes, and their interactions might not be acquired in STRING yet. These findings highlighted their importance, which led us to focus on the blue module. We performed GO functional enrichment analysis and KEGG pathway enrichment analysis on the key genes from the blue module to facilitate future research. Functional annotation is mainly related to the self-renewal and proliferation characteristics of stem cells. The pathway enrichment suggested that the 13 key genes in the cell cycle pathway term were most likely a functional gene set that affects tumor stemness by regulating the cell cycle.

Survival curves were generated to validate the prognostic value of these 13 key genes of the cell cycle signaling pathway in LUADs. In the K–M plots, all 13 cell cycle over-expressing groups were significantly associated with poor prognoses. This result was consistent with the fact that their module was positively correlated with mRNAsi. The PPI network showed significantly more interactions than the expected number assessed according to the genome. This indicated that the proteins of this gene set might have close biological connections and perform biological functions as a whole. The pan-cancer gene expression profile showed that the key genes were regulated in many other cancers, which indicates the stemness gene set may share similarities in the pan-cancer scale.

Given that various organs in the human body are differentiated from pluripotent stem cells, dedifferentiated CSCs inherit some of the common characteristics of stem cells, conversely. The expression of key genes changed significantly with tumor progression in the violin plots (Appendix B), indicating that cancer stem cell features may be involved in the deterioration of LUAD, potentially. Eight of the 13 genes have been reported to be associated with the characteristics of cancer stem cells; however, none were reported for LUADs.

The differential expression of CCNB1 was reported in a gene chip profile, between the CD133+ and CD133- subpopulations in the SW480 colon adenocarcinoma cell line [38]. The knockdown of BUB1 in the MDA-MB-231 breast cancer cell line reduced the CSC potential [39]. The depletion of BUB1B in embryonic stem cells (ESCs) compromised self-renewal and led to consequent differentiation by the mechanism of DNA damage/genome instability, activating p53, and culminating in ESC differentiation [40]. CDC20 was usually enriched in CD44+ prostate CSCs [41]. Downregulation of PLK1 protein enhanced the drug-resistance of temozolomide (TMZ) in CD133+ stem-like glioma cell lines, while G2/M arrest was induced significantly [42]. TTK was identified as one of the angiogenic modulators in a robust ESC-based vascular differentiation assay, reducing tumor growth, vascular density, and improving lung carcinoma survival in vivo [43].

In glioma stem-like cells (GSCs), the MTFR2-dependent regulation of TTK was validated for a crucial role in maintaining GSCs in gliomas [44]. High expression of MCM2 in clinical samples was reported with CSC marker-positive breast cancer cells, and the MCM2-targeted therapeutic strategy, together with Hph-1-gp70 treatment to induce DNA damage, were regarded as a potential therapy for the eradication of stem-like cells from breast cancer tissue [45]. Checkpoint kinase1 (CHEK1), one of the DNA-damage checkpoint proteins, was reported reducing the stem cell population in ovarian cancer, triggered by its inhibitor (LY2603618) [46]. In hepatocellular carcinoma (HCC), downregulation of CHEK1 by silencing LINC01224 or elevating miR-330-5p could inhibit stemness, by reducing the expression of CSC biomarkers (OCT4, CD133 and SOX2) [47]. The genes of CDC45, ESPL1, CCNA2, MCM6, and ORC1 have not been reported on the CSC issues in our survey.

We also validated the key genes from the blue module in GSE21656. The only intersection was TRAIP, which has been reported as a novel regulator of H2B monoubiquitination in DNA damage response and cancer development in LUAD [48]. Having only one gene alignment may be of low utility; however, the number of genes detected by the microarray was limited, and the cell line of this array was H460, which is a large cell lung cancer cell line, while still being a NSCLC, the histological differences compared with lung adenocarcinoma should also be considered.

## 5. Conclusions

In summary, we have discovered 13 key genes of the cell cycle pathway, which play important roles in LUAD stemness features. The validations showed that these genes could be useful for outlining the prognosis of LUAD patients. All of them have been previously ignored, and have the potential to become additional biomarkers for LUAD. However, our conclusions are based on an in silico approach, and further investigation of these genes could lead to novel insights into the potential associations of CSCs with a LUAD prognosis.

## Figures and Tables

**Figure A6:**
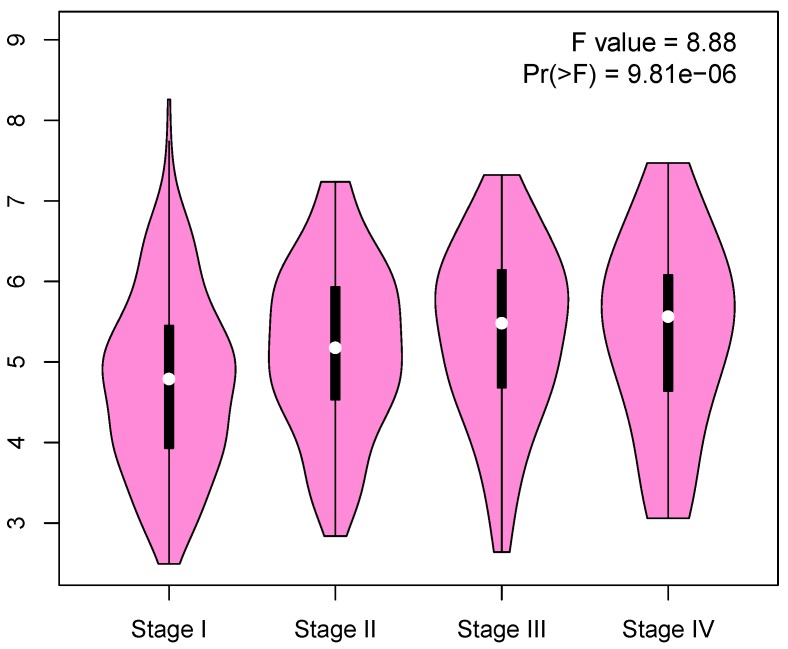
CCNB1.

**Figure A7:**
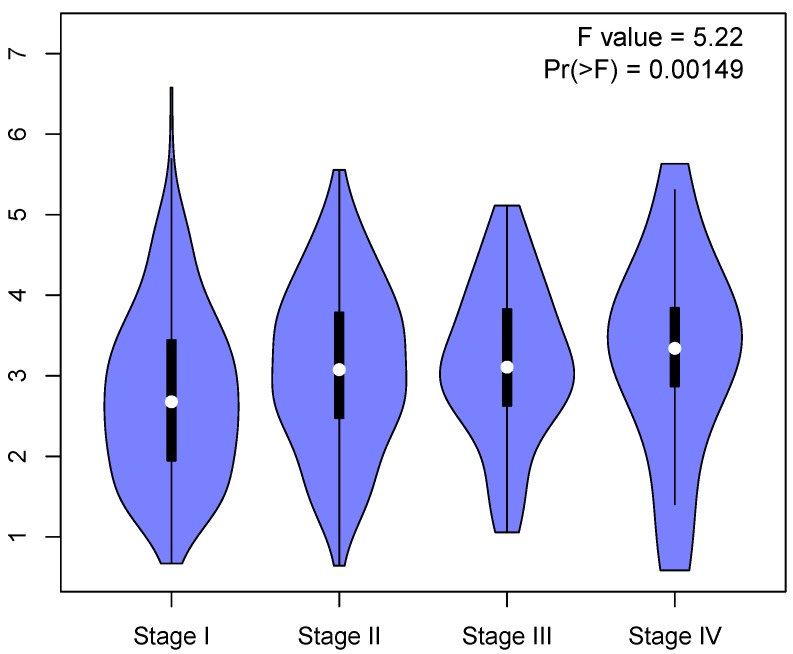
BUB1.

**Figure A8:**
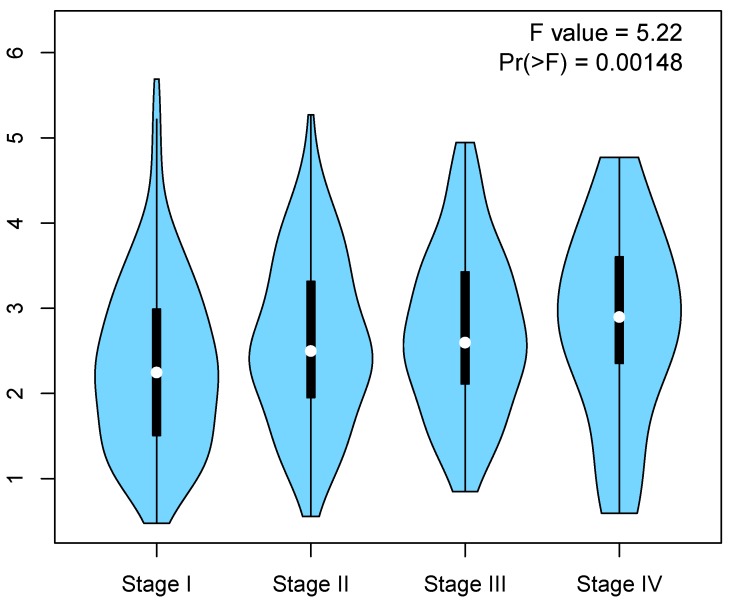
BUB1B.

**Figure A9:**
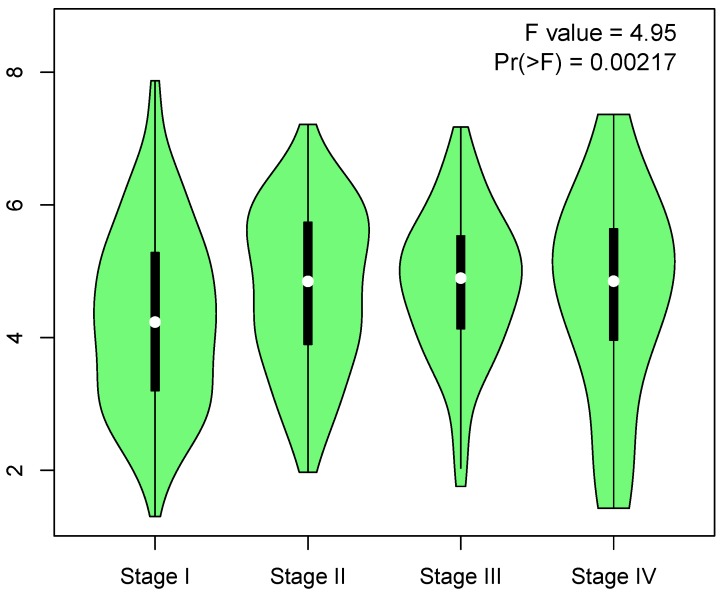
CDC20.

**Figure A10:**
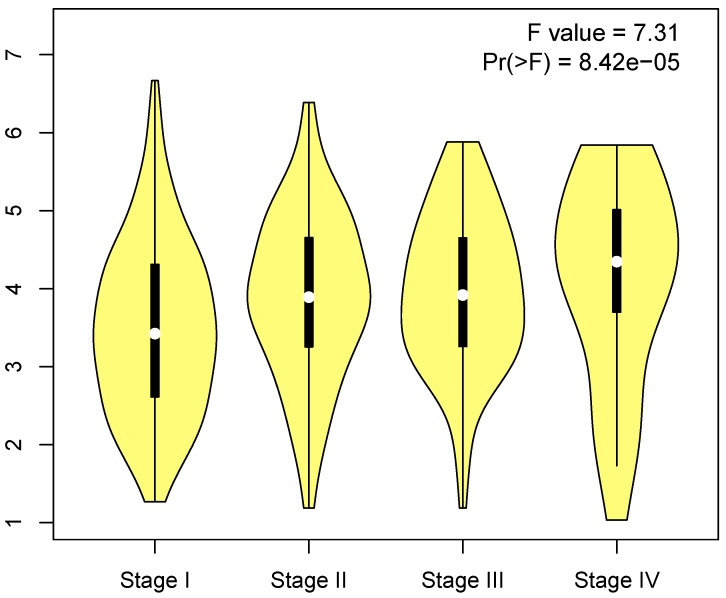
PLK1.

**Figure A11:**
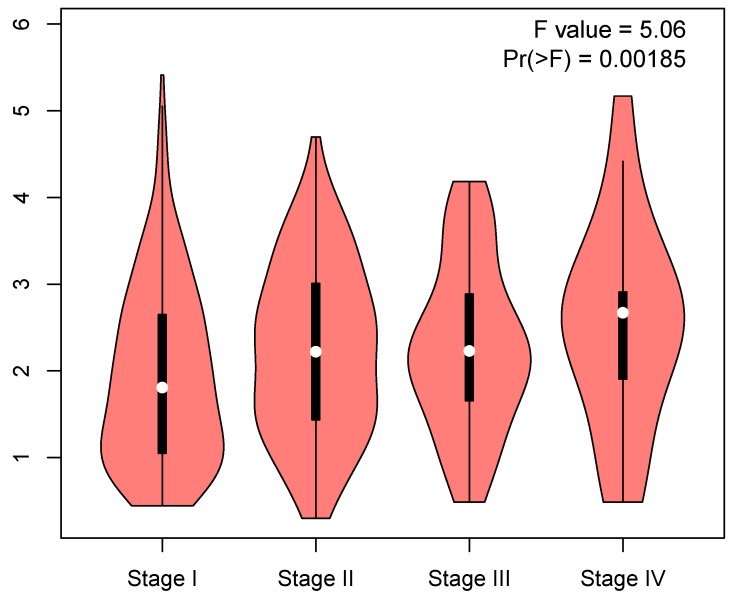
TTK.

**Figure A12:**
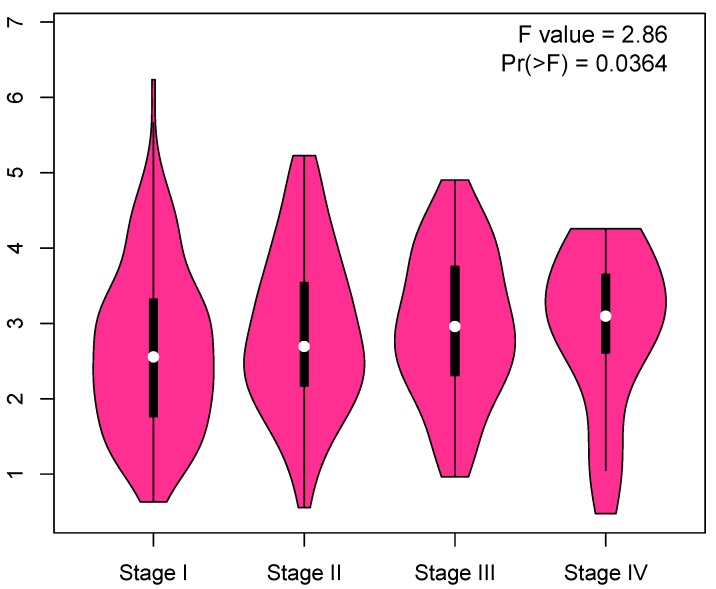
CDC45.

**Figure A13:**
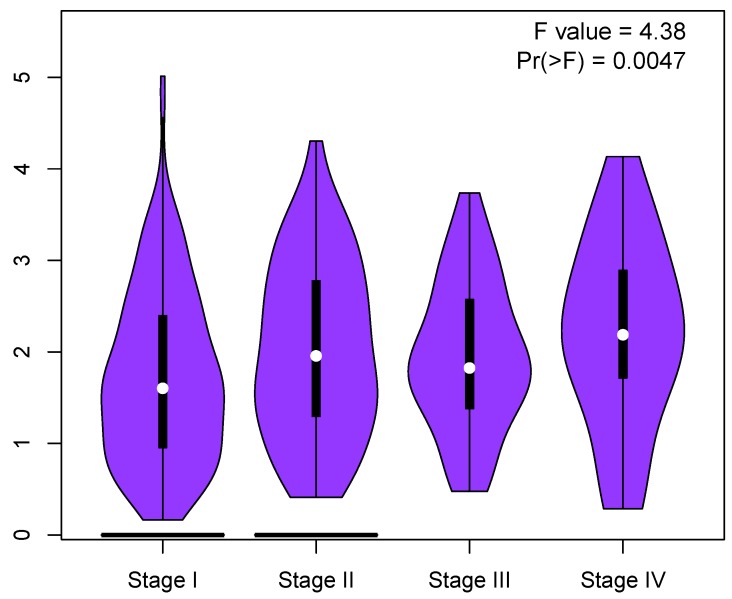
ESPL1.

**Figure A14:**
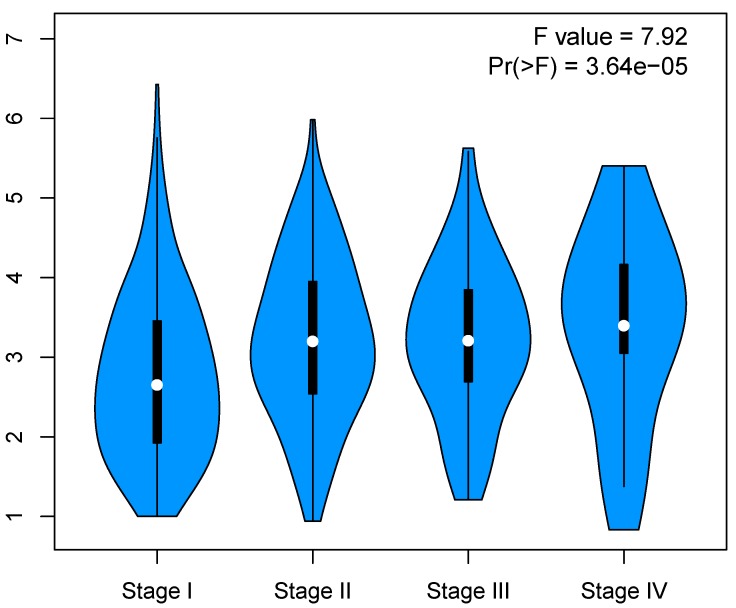
CCNA2

**Figure A15:**
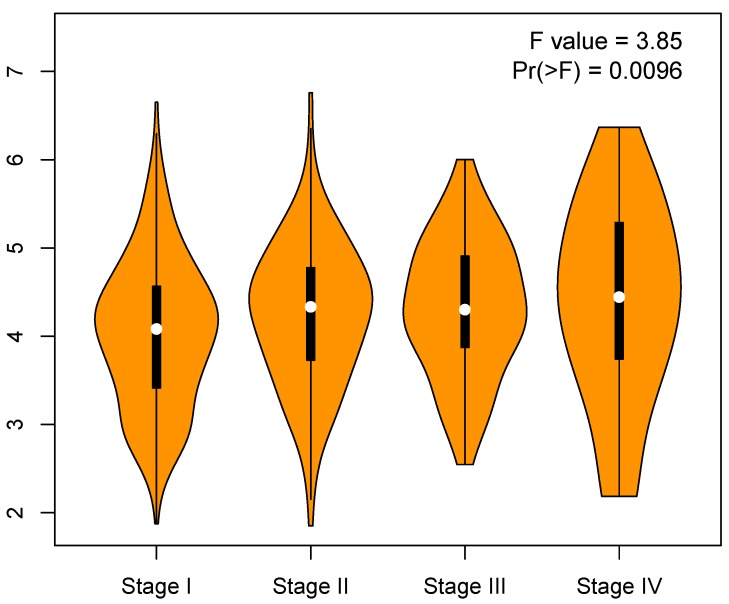
MCM6.

**Figure A16:**
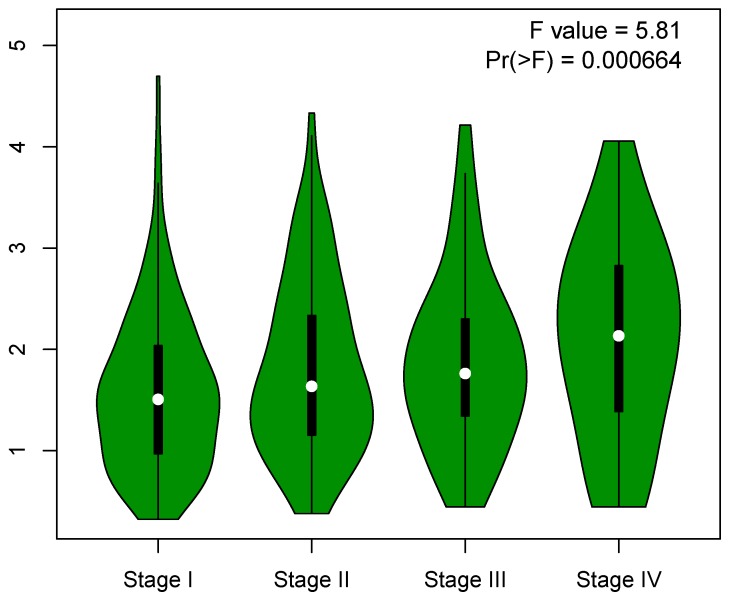
ORC1.

**Figure A17:**
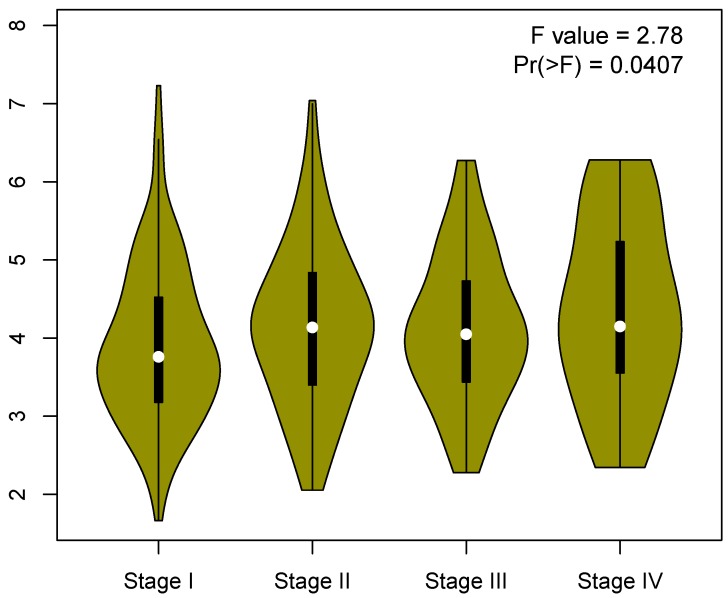
MCM2.

**Figure A18:**
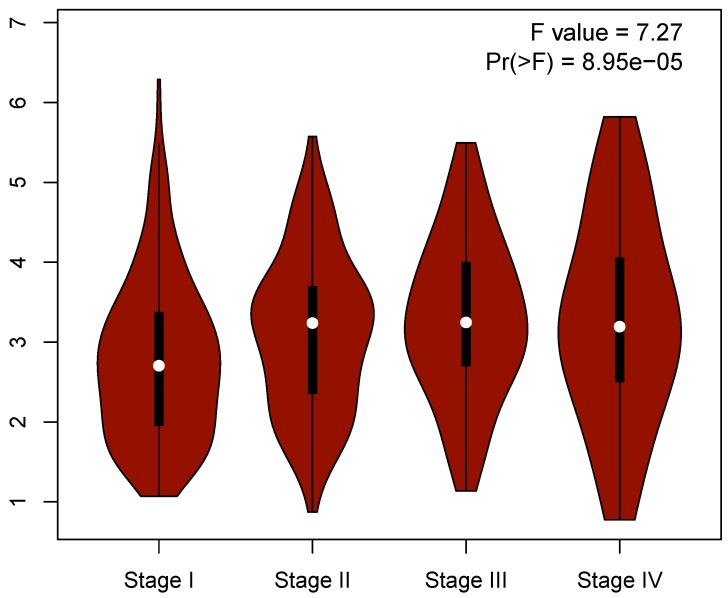
CHEK1.

## Appendix A. The Steps to Draw the Dendrograms

WGCNA was performed to construct gene co-expression networks in modules with biological significance and to extract better functional gene sets associated with stemness in LUADs. The samples with an outlier of gene expression (above the red line) are eliminated (Figure A1). The heat map shows the global overview of the mRNAsi and the EREG-mRNAsi expression in screened cases (Figure A2). We selected the appropriate powervalue = 4, taking both the scale-free correlation coefficient and mean connectivity into consideration (Figure A3). Identification of scale-free networks for LUAD used the power value. The branches of the cluster dendrogram correspond to the 17 different gene modules. Each piece of the leaves on the cluster dendrogram corresponds to a gene (Figure A4). We screened the modules with a standard of a minimum size of 50 genes for module merging. Close modules were merged at the DissThres of 0.25 (Figure A5).

## Appendix B. Correlation between Cell Cycle Key Gene Expression and Tumor Stages in LUAD Patients

Violin plots show the expression levels of key genes associated with pathological stage, performed by GEPIA (Figure A6, Figure A7, Figure A8, Figure A9, Figure A10, Figure A11, Figure A12, Figure A13, Figure A14, Figure A15, Figure A16, Figure A17 and Figure A18), which indicated that all of the 13 stemness key genes might be involved in tumor progression. The Pr(>F) stands for the *p*-value calculated bases on the *F*-value, and Pr(>F) < 0.05 was considered statistically significant.
